# Frailty in Diabetic Population: A Study From Northern India

**DOI:** 10.7759/cureus.68494

**Published:** 2024-09-02

**Authors:** Samyak Golchha, Shankerdeep Sondhi, Sunita Gupta, Ashank Goel

**Affiliations:** 1 General Medicine, Maharishi Markandeshwar Institute of Medical Sciences & Research, Mullana, IND

**Keywords:** diabetic frailty, diabetes mellitus in elderly, geriatric medicine, edmonton frail scale, ageing and frailty

## Abstract

Introduction

Frailty, a key issue in geriatric health, signifies heightened vulnerability due to the decline in various physiological systems, exacerbated by conditions such as diabetes. Diabetes and frailty together lead to significant disabilities and higher mortality, necessitating early screening and targeted interventions. The relationship between frailty and diabetes remains under-researched, prompting this study to explore their association in individuals over 50 years of age using the Edmonton Frail Scale (EFS).

Methods and materials

The study was an observational cross-sectional study conducted at MM Institute of Medical Sciences & Research (MMIMSR), Mullana, India, among 102 diabetic and 100 non-diabetic individuals aged more than 50 years, with data collected through interviews using a pre-validated proforma. Frailty was assessed using the EFS, categorizing patients into fit, vulnerable, and various levels of frailty based on their scores.

Results

The study found a higher prevalence and severity of frailty among diabetic individuals (61.8%) compared to non-diabetics (29%), with frailty being more pronounced across all age groups and both genders in diabetics. The severity of frailty increased with the duration of diabetes but showed no significant correlation with glycemic control (HbA1c).

Strengths and limitations

The study prospectively collected data, including middle-aged participants starting from age 50, and uniquely used the EFS to assess frailty in diabetic patients, excluding those with other chronic diseases (end-stage renal disease (ESRD), malignancy, etc.). However, limitations included a small sample size, recruitment from a single institution in India, and some EFS questions being less relevant to the Indian diabetic population.

Conclusion

The study found a 61.8% prevalence of frailty in diabetics compared to 29% in non-diabetics, with frailty being more severe and positively correlated with the duration of diabetes but not with glycemic control (HbA1c).

## Introduction

Aging is marked by a gradual decline in functional abilities due to physiological, social, psychological, and biological changes. This irreversible process affects individuals, families, and society, leading to general functional impairments and increased susceptibility to age-related illnesses, such as diabetes, cancer, hypertension, kidney diseases, Parkinson’s disease, dementia, cardiovascular diseases, and arthritis. Elderly individuals also face social isolation, which is linked to chronic conditions causing speech, vision, and hearing impairments. The risk of death from non-communicable chronic and degenerative diseases now surpasses that of communicable diseases.

Frailty is a major concern in geriatric health, characterized by increased vulnerability to aging's negative effects on various physiological systems. It results in a decline in reserve capacity, making individuals more susceptible to functional impairments and higher mortality from minor stressors. Addressing frailty requires focusing on biological age rather than chronological age as factors such as aging, poverty, illiteracy, poor health, and co-morbidities contribute to higher frailty prevalence.

Diabetes complications, such as retinopathy, cardiovascular disease, renal failure, and peripheral vascular disease, increase with disease duration, leading to functional decline and disability. Frailty is a significant complication in elderly individuals with diabetes, necessitating early intervention and prevention plans to improve outcomes. Frailty in diabetic individuals manifests earlier and increases the risks of hospitalizations and mortality. Physicians should screen for frailty early in diabetic patients to improve care and outcomes. Enhanced healthcare budgets and preventive programs are needed [1,2].

Literature review showed that research to date on frailty has primarily focused on the general population, with limited studies on individuals with diabetes mellitus. Despite the rising prevalence of diabetes in India, there was a lack of research on the relationship between frailty and diabetes. This study aimed to assess the association between frailty and diabetes mellitus in individuals over 50 years of age using the Edmonton Frail Scale (EFS).

## Materials and methods

Material and methods

A sample size of 202 participants was taken, comprising 102 diabetics (cases) and 100 non-diabetics (controls). The inclusion criteria for the study were diabetic subjects aged over 50 years, as per the American Diabetes Association (ADA) guidelines [3], and non-diabetic subjects matched for age and sex. Exclusion criteria ruled out elderly subjects with Parkinson’s disease, previous stroke, hemodynamically unstable conditions, or documented terminal illnesses, such as malignancy and ESRD.

Methodology 

The study was an observational cross-sectional study conducted in the Department of General Medicine at MMIMSR, Mullana. Participants were informed about the study's purpose and methods, and consent was obtained in their preferred language, ensuring confidentiality. A thorough history of diabetes duration, comprehensive clinical examinations, and routine investigations (including random blood sugar (RBS), complete blood count (CBC), renal function test (RFT), liver function test (LFT), HbA1c, ECG, and chest X-ray) were conducted. Data were collected through interviews using a pre-validated proforma in the participants' preferred language or translated as needed. All subjects, both cases and controls, were divided into three age groups: 51-60 years, 61-70 years, and 71 years and above.

**Figure 1:**
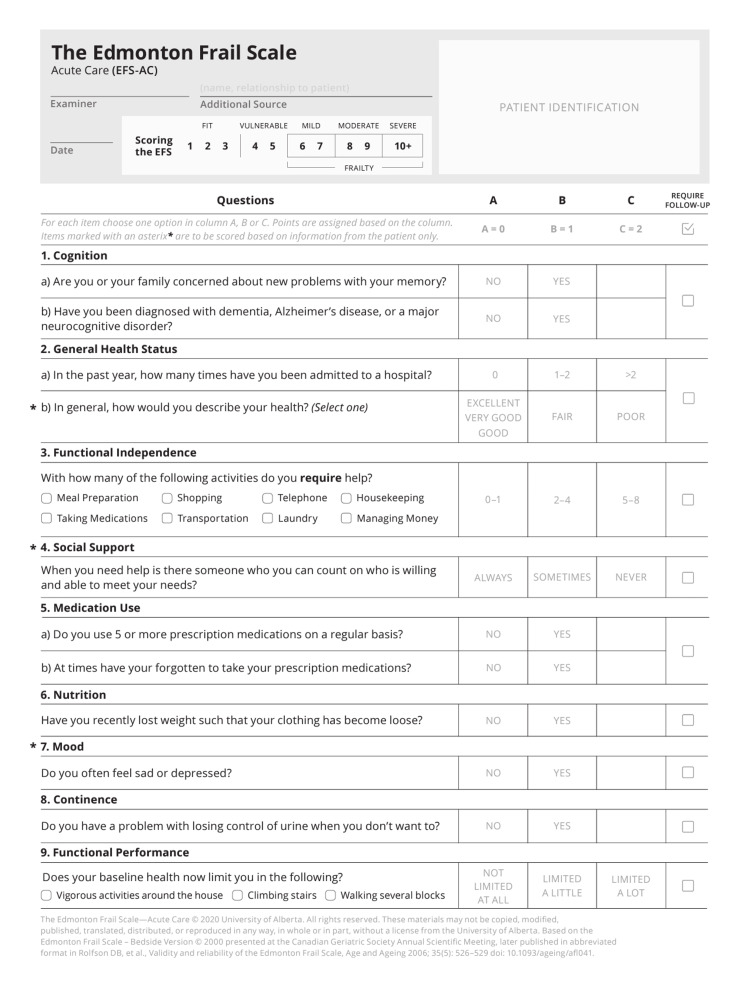
The Edmonton Frail Scale Acute Care (EFS-AC) The Edmonton Frail Scale—Acute Care © 2020 University of Alberta. All rights reserved. These materials may not be copied, modified, published, translated, distributed, or reproduced in any way, in whole or in part, without a license from the University of Alberta. Based on the Edmonton Frail Scale – Bedside Version © 2000 presented at the Canadian Geriatric Society Annual Scientific Meeting, later published in abbreviated format in Rolfson DB, et al., Validity and reliability of the Edmonton Frail Scale, Age and Ageing 2006; 35(5): 526–529 doi: 10.1093/ageing/afl04

Frailty was assessed using the EFS-Acute Care (EFS-AC) [4], a questionnaire-based tool evaluating various dimensions of health and cognitive function (Figure 1). Patients were categorized as fit, vulnerable, and frail. The severity of frailty among those identified as frail was further classified based on their scores: fit (1-3), vulnerable (4-5), mild frailty (6-7), moderate frailty (8-9), and severe frailty (10 or higher).

Statistical analysis

The collected data was digitized using MS Excel (Microsoft® Corp., Redmond, WA) and analyzed using Statistical Product and Service Solutions (SPSS, version 20; IBM SPSS Statistics for Windows, Armonk, NY). Descriptive statistics, including frequency, percentage, ratio, minimum, maximum, mean, and standard deviation, were used to describe data dispersion. Observations on frailty among diabetic and non-diabetic patients were presented both tabularly and graphically. Categorical data were assessed and compared based on frequency and percentages. Numerical data were analysed using descriptive statistics and categorised by frailty severity. The chi-square test or Fisher's exact test was used to compare frailty status between groups. The Edmonton Frail Scale (EFS) scores were compared using the Mann-Whitney U test. Spearman's rho correlation coefficient examined the relationship of EFS scores with HbA1c, RBS, and disease duration among diabetic patients. Multiple logistic regression identified predictive factors for EFS among diabetic patients, with a statistical significance level set at p<0.05.

Ethical justification

During the study, the treatment of the patients was not hampered or delayed. Written, voluntary, and informed consent was obtained from each patient in a language they could completely understand. Every patient had the freedom to withdraw from the study at any time without needing to provide a justification. Patient treatment was not affected if a patient decided not to participate in the study; routine therapy continued to be provided. The patients were not financially burdened by the study as all expenses were covered by the researchers or institution. The Institute's Ethics Committee (IEC) approved the study before it started (Letter- IEC No. 2514).

## Results

In our study, we included a total of 202 participants, with 102 having diabetes (cases) and 100 without diabetes (controls). The average age of the diabetes group was 63.48 ± 7.90 years (males: 63.89; females: 63.12), and for the non-diabetes group, it was 64.13 ± 7.39 years (males: 63.88; females: 64.38). The diabetes group included 47 male and 55 female participants, while the non-diabetes group had 50 male and 50 female participants. Both groups had similar distributions of age and gender.

Table 1 shows the average scores for each component of the EFS scale. The data show that diabetic cases had statistically significant differences from non-diabetic controls (p<0.05) in several areas: poor general health, greater functional dependence, higher medication use, lower mood, urinary incontinence, and impaired functional performance. However, there were no statistically significant differences (p>0.05) between the two groups in terms of cognition, social support, and nutrition.

**Table 1:** Comparison of various domains of the Edmonton Frail Scale (EFS) among cases (diabetics) and controls (non-diabetics) SD - Standard Deviation; IQR - Inter-Quartile range; NS - non-significant (p>0.05) ; S - Significant (p<0.05)

Domain		Mean ± SD	Min. - Max.	Median (IQR)	Mode	p-value
Cognition	Diabetic (Cases) (n-102)	0.16 ± 0.42	0 - 2	0.0 (0.0- 0.0)	0	0.181^NS^
	Non-diabetic (Controls) (n-100)	0.08 ± 0.27	0 - 1	0.0 (0.0 - 0.0)	0	
General Health	Diabetic (Cases) (n-102)	2.18 ± 0.89	0 - 4	2.0 (2.0 - 3.0)	2	0.015^S^
	Non-diabetic (Controls) (n-100)	1.86 ± 0.84	0 - 4	2.0 (1.0 - 2.0)	2	
Functional dependence	Diabetic (Cases) (n-102)	0.89 ± 0.73	0 - 2	1.0 (0.0 - 1.0)	1	0.009^S^
	Non-diabetic (Controls) (n-100)	0.63 ± 0.70	0 - 2	0.5 (0.0 - 1.0)	0	
Social support	Diabetic (Cases) (n-102)	0.03 ± 0.17	0 - 1	0.0 (0.0 - 0.0)	0	0.980^NS^
	Non-diabetic (Controls) (n-100)	0.03 ± 0.17	0 - 1	0.0 (0.0 - 0.0)	0	
Medication use	Diabetic (Cases) (n-102)	0.76 ± 0.54	0 - 2	0.0 (0.0 - 1.0)	1	0.001^S^
	Non-diabetic (Controls) (n-100)	0.39 ± 0.49	0 - 1	0.0 (0.0 - 1.0)	0	
Nutrition	Diabetic (Cases) (n-102)	0.39 ± 0.49	0 - 1	0.0 (0.0 - 1.0)	0	0.359^NS^
	Non-diabetic (Controls) (n-100)	0.33 ± 0.47	0 - 1	0.0 (0.0 - 1.0)	0	
Mood	Diabetic (Cases) (n-102)	0.38 ± 0.48	0 - 1	0.0 (0.0 - 1.0)	0	0.029^S^
	Non-diabetic (Controls) (n-100)	0.24 ± 0.42	0 - 1	0.0 (0.0 - 0.0)	0	
Continence	Diabetic (Cases) (n-102)	0.25 ± 0.43	0 - 1	0.0 (0.0 - 1.0)	0	0.001^S^
	Non-diabetic (Controls) (n-100)	0.05 ± 0.21	0 - 1	0.0 (0.0 - 0.0)	0	
Functional performance	Diabetic (Cases) (n-102)	1.36 ± 0.62	0 - 2	1.0 (1.0 - 2.0)	1	0.001^S^
	Non-diabetic (Controls) (n-100)	0.98 ± 0.61	0 - 2	1.0 (1.0 - 1.0)	1	

Table 2 shows prevalence of frailty among diabetics is 61.8 % and 29 % among non-diabetic subjects.

**Table 2:** Prevalence of frailty in our study S - Significant (p<0.05)

Prevalence of frailty among the study subjects (Frailty status)	Diabetic (Cases) (n-102)		Non-diabetic (Controls) (n-100)		p-value
	F	%	F	%	
Fit	12	11.8	34	34.0	0.001^S^
Vulnerable	27	26.5	37	37.0	
Frailty	63	61.8	29	29.0	

Table 3 shows that, among diabetic cases, 63 individuals were frail: 29 (28.4%) with mild frailty, 21 (20.6%) with moderate frailty, and 13 (12.7%) with severe frailty. In contrast, among non-diabetic controls, 29 individuals were frail: 14 (14%) with mild frailty, 14 (14%) with moderate frailty, and 1 (1%) with severe frailty. Diabetic cases had statistically significantly more severe frailty compared to controls (p=0.001).

**Table 3:** Distribution of frailty according to severity of frailty S - Significant (p<0.05)

Extent of Frailty	Diabetic (Cases) (n-63)		Non-diabetic (Controls) (n-29)		p-value
	F	%	F	%	
Mild Frailty	29	46.0	14	48.3	0.001^S^
Moderate Frailty	21	33.3	14	48.3	
Severe Frailty	13	20.6	1	3.4	

**Figure 2:**
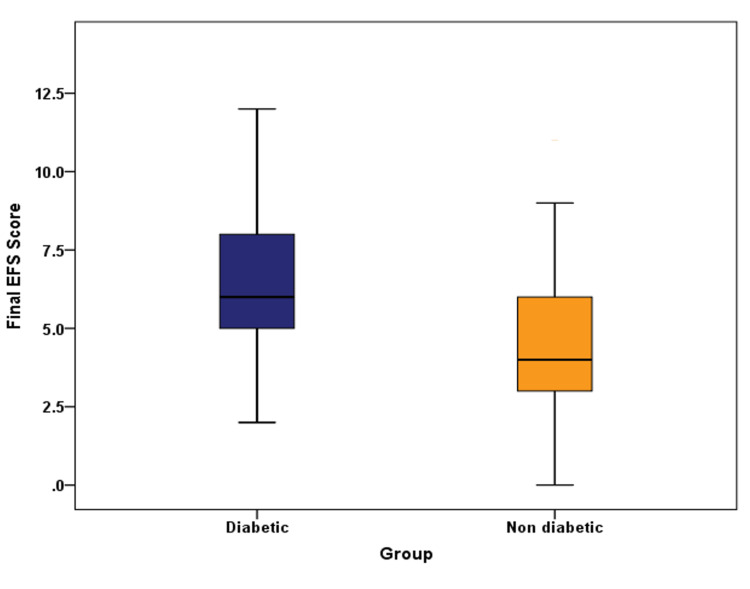
Comparison of the final Edmonton Frail Scale (EFS) score among cases and controls

The mean final EFS score, as shown in Figure 2, was significantly higher in diabetic cases (6.41 ± 2.50) compared to non-diabetic controls (4.59 ± 2.33) (p=0.001).

Table 4 demonstrates that frailty was significantly higher among cases compared to controls across all age groups.

**Table 4:** Correlation of the EFS score with age among cases and controls S - Significant (p<0.05)

Age (years)	Diabetic (Cases) (n-102)			Non-diabetic (Controls) (n-100)			p-value
	N	Mean ± SD	Median (IQR)	N	Mean ± SD	Median (IQR)	
51 - 60	43	5.84 ± 2.42	6.00 (4.00 - 7.00)	39	4.00 ± 2.11	4.00 (3.00 - 5.00)	0.001^S^
61 - 70	41	6.49 ± 2.52	6.00 (5.00 - 8.00)	40	4.70 ± 2.28	5.00 (3.00 -6.00)	0.002^S^
> 70	18	7.61 ± 2.33	8.00 (5.75 - 9.25)	21	5.48 ± 2.58	5.00 (4.00 - 7.50)	0.015^S^

Frailty, assessed using the EFS scores, was compared between diabetic and non-diabetic individuals of both genders. Diabetic individuals exhibited statistically significantly higher frailty (p<0.05) in both genders.

**Figure 3:**
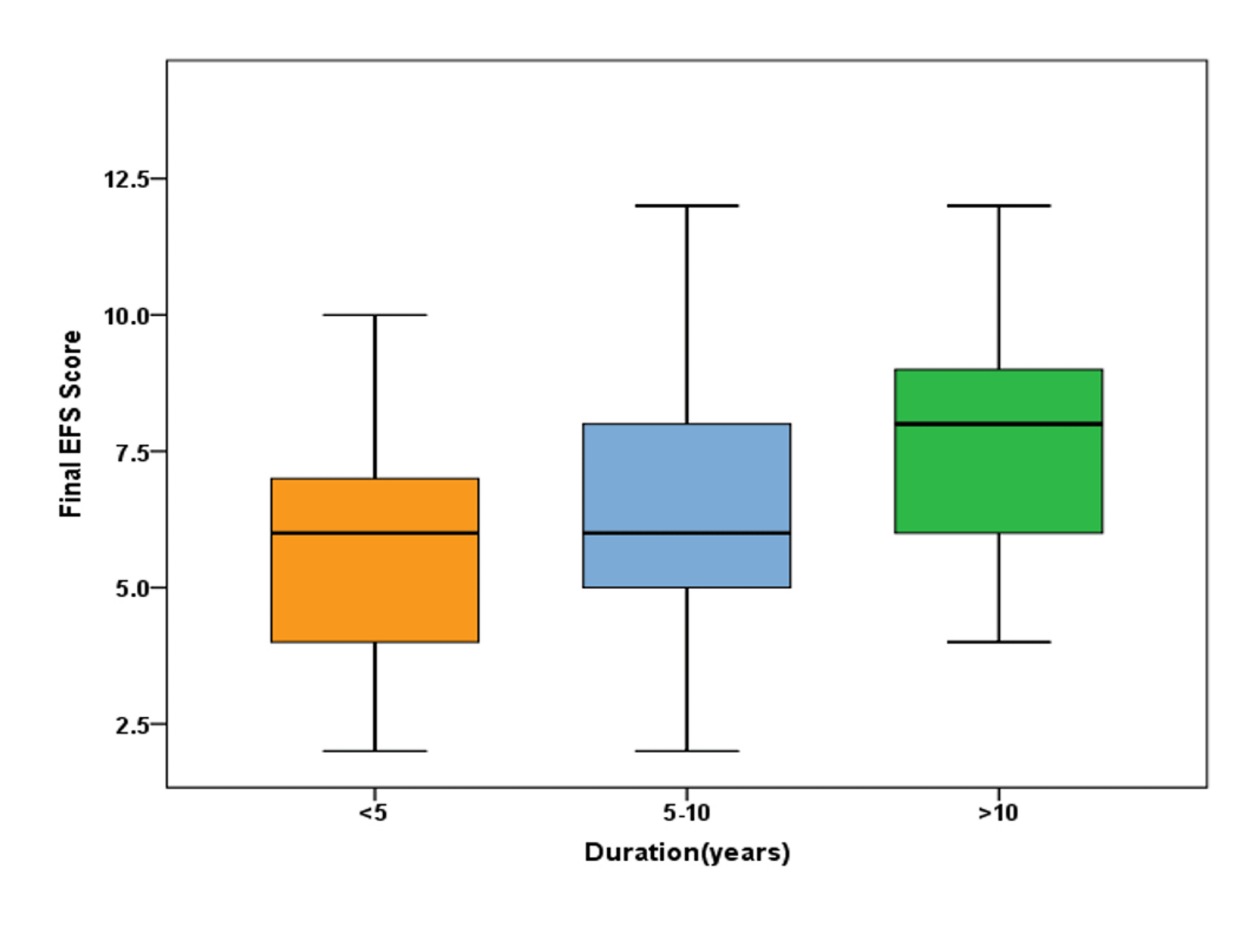
Correlation of the EFS score with the duration of diabetes among diabetics

Figure 3 illustrates a positive correlation between the duration of diabetes and the mean EFS score, which suggests an increase in frailty with the duration of diabetes (p=0.012).

## Discussion

In this study, we enrolled 202 subjects, consisting of 102 diabetic cases and 100 age and gender-matched non-diabetic controls. The average age of the diabetes group was 63.48 ± 7.90 years (males: 63.89; females: 63.12), and for the non-diabetes group, it was 64.13 ± 7.39 years (males: 63.88; females: 64.38).

We included both middle-aged (50-60 years) and the elderly population (>60 years) to investigate whether frailty in the diabetic population begins earlier compared to the general population. Consequently, the mean age of participants in our study was comparatively lower than that in other studies [5]. In contrast, studies conducted by Park et al. [6], Jung et al. [7], and Cruz et al. [8] focused on elderly populations with a mean age exceeding 70 years.

Table 4 shows that frailty onset begins in the middle-aged group (51-60 years) among the diabetic population, in contrast to the non-diabetic population. This suggests a link between frailty and diabetes, where diabetes serves as an added contributing factor that accelerates biological aging.

Similarly, studies by Ottenbacher et al. [9] and Hubbard et al. [10] observed that individuals with diabetes exhibited frailty at a younger age (81.3 years) compared to those without diabetes (83.3 years), despite having comparable levels of frailty.

In our study, responses from subjects were documented using a structured proforma, and mean scores for each domain of the EFS-AC [3] were calculated and compared between diabetic cases and non-diabetic controls.

Cognitive impairment in the form of memory loss was present in 13.7 % of the diabetic population, and two subjects had severe dementia, but it was not observed in the non-diabetic population.

Table 1 reveals that cognitive impairment was higher in diabetic subjects compared to non-diabetic controls. Similarly, Bu et al. [11] found a strong association between frailty and cognitive decline in their study in China.

Diabetic individuals exhibited significantly poorer general health in terms of recurrent hospital admissions and self-reported poor health status.

Additionally, 67.6 % of the diabetic population has functional dependencies in the form of the inability to do routine household activities such as meal preparation, shopping, laundry, housekeeping, etc.

Social support scores were nearly identical between diabetics and non-diabetics (0.03 ± 0.17; p=0.980), likely due to the common practice of joint family living arrangements in India. In Western countries, diabetics who live alone often lack psychological and social support during stressful situations [12]. However, this situation is uncommon in India.

Medication use was significantly higher among diabetics, corroborating findings by Gutiérrez-Valencia et al. [13], who observed a significant correlation between polypharmacy and frailty in their systematic review and meta-analysis.

Nutritional status differences in the form of weight loss were not statistically significant in our study. In India, many diabetic individuals are obese, and weight loss was used as a criterion for nutritional assessment in the EFS scale. Diabetes mellitus, as a chronic wasting disease, can lead to weight loss and reduced waist circumference, as noted by Bu et al. [11].

Park et al. [6] reported that diabetes is associated with reduced skeletal muscle strength and quality, which may contribute to the development of physical disabilities in older adults with diabetes.

Depression was significantly more prevalent among diabetic cases (38.25 %, mean score = 0.38 ± 0.48) compared to controls (24 %, mean score = 0.24 ± 0.42; p=0.029). Numerous studies have demonstrated that depression and frailty share similar pathophysiological mechanisms, and depressive symptoms can exacerbate frailty by reducing social activity [14]. The prevalence of depression in diabetic patients is approximately 15%, nearly double that of non-diabetic individuals [15].

Urinary incontinence was significantly more common in diabetic cases (25%, mean score = 0.25 ± 0.43) compared to controls (5%, mean score = 0.05 ± 0.21; p=0.001).

Diabetic cases also exhibited significantly greater impairment in functional performance in terms of decreased baseline health in doing vigorous household activities, climbing stairs, and walking several blocks (mean score = 1.36 ± 0.62) compared to controls (mean score = 0.98 ± 0.61; p=0.001). Similar findings were reported by Yang et al. [16] and Carneiro et al. [17] in their respective studies.

The mean frailty score in diabetics (6.41 ± 2.50) was significantly higher than in the non-diabetic group (4.59 ± 2.33) (p=0.001).

Comparing frailty between cases and controls in both genders revealed that both male and female diabetics had significantly greater frailty levels than their non-diabetic counterparts (p<0.05). Among non-diabetics, men exhibited higher frailty than women, whereas among diabetics, women showed higher frailty levels but the difference was not statistically significant.

Previous studies have found a significant association between frailty and gender (female) in the general population; however, our study did not observe this association. This discrepancy may be due to various factors such as educational status, financial independence, and marital status, as women today are more educated and financially independent.

Age may be the confounding factor for Frailty, but in our study, both the genders in cases and control groups had similar mean ages.

A significant increase in frailty was observed with increasing duration of diabetes (p=0.012). In a study conducted in Taiwan, Li et al. [18] reported that diabetic participants who were frail tended to be older and had a longer duration of diabetes.

Our study also assessed the correlation between frailty and glycemic control by comparing mean EFS scores with HbA1c levels. We found no statistically significant difference in mean EFS scores across various HbA1c categories (p=0.266), indicating no correlation between glycemic control (HbA1c) and frailty among diabetic patients. In contrast, García-Esquinas et al. [19] identified a significant association between elevated glycated HbA1c levels and increased odds of frailty, with an odds ratio of 1.48 (95% CI: 1.20-1.81) for each 1% increase in HbA1c.

## Conclusions

Our study revealed a significantly higher prevalence of frailty among the diabetic population (61.8%) compared to the non-diabetic population (29%). This stark difference underscores the profound impact that diabetes has on physical health and resilience, affecting individuals across various age groups. The higher prevalence of frailty among diabetic patients emphasizes the critical need for heightened awareness and proactive management strategies to mitigate the progression of frailty in this vulnerable group.

Moreover, our research demonstrated that the severity of frailty was markedly greater in diabetic patients, with 20.6% experiencing severe frailty, in contrast to only 3.4% of non-diabetic patients. This finding, coupled with the observed positive correlation between frailty and the duration of diabetes, highlights the cumulative toll that prolonged diabetes can take on an individual's functional status. The study emphasizes the necessity for healthcare providers to routinely assess frailty in diabetic patients to identify those at risk and to tailor interventions that can prevent or delay the onset of severe frailty. Given the implications for patient care, future studies should aim to include larger and more diverse populations to improve the generalizability of these findings. A deeper understanding of the relationship between diabetes and frailty, along with the prevalence of frailty in diabetic patients, could lead to the development of more effective treatment strategies and an overall improvement in quality of life. Integrating frailty assessments into routine clinical practice, alongside standard screenings for diabetic complications, is essential. Additionally, implementing educational programs focused on nutrition, muscle strength, and the management of modifiable risk factors could play a crucial role in preventing disability among pre-frail and frail diabetic individuals.

## References

[1] Wong E, Backholer K, Gearon E, Harding J, Freak-Poli R, Stevenson C, Peeters A (2013). Diabetes and risk of physical disability in adults: a systematic review and meta-analysis. Lancet Diabetes Endocrinol.

[2] Narayan KM, Boyle JP, Thompson TJ, Sorensen SW, Williamson DF (2003). Lifetime risk for diabetes mellitus in the United States. JAMA.

[3] Cornell S (2017). Comparison of the diabetes guidelines from the ADA/EASD and the AACE/ACE. J Am Pharm Assoc (2003).

[4] Rolfson DB, Majumdar SR, Tsuyuki RT, Tahir A, Rockwood K (2006). Validity and reliability of the Edmonton frail scale. Age Ageing.

[5] Al-Ali SA, AlJabr QM, Alramadhan ZT, Algharrash Z, Alyousif AJ, Alessa AN, AlButayan HA (2021). Screening of diabetic patients for frailty with the frail scale: a comparison with the Fried’s phenotype criteria in Saudi Arabia. Int J Diabetes Clin Res.

[6] Park SW, Goodpaster BH, Strotmeyer ES (2006). Decreased muscle strength and quality in older adults with type 2 diabetes: the health, aging, and body composition study. Diabetes.

[7] Jung HW, Kim SW, Ahn S (2014). Prevalence and outcomes of frailty in Korean elderly population: comparisons of a multidimensional frailty index with two phenotype models. PLoS One.

[8] Cruz DT, Vieira MT, Bastos RR, Leite IC (2017). Factors associated with frailty in a community-dwelling population of older adults. Rev Saude Publica.

[9] Ottenbacher KJ, Graham JE, Al Snih S, Raji M, Samper-Ternent R, Ostir GV, Markides KS (2009). Mexican Americans and frailty: findings from the Hispanic established populations epidemiologic studies of the elderly. Am J Public Health.

[10] Hubbard RE, Andrew MK, Fallah N, Rockwood K (2010). Comparison of the prognostic importance of diagnosed diabetes, co-morbidity and frailty in older people. Diabet Med.

[11] Bu F, Deng XH, Zhan NN (2023). Development and validation of a risk prediction model for frailty in patients with diabetes. BMC Geriatr.

[12] Fried LP, Tangen CM, Walston J (2001). Frailty in older adults: evidence for a phenotype. J Gerontol A Biol Sci Med Sci.

[13] Gutiérrez-Valencia M, Izquierdo M, Cesari M, Casas-Herrero Á, Inzitari M, Martínez-Velilla N (2018). The relationship between frailty and polypharmacy in older people: a systematic review. Br J Clin Pharmacol.

[14] Vaughan L, Corbin AL, Goveas JS (2015). Depression and frailty in later life: a systematic review. Clin Interv Aging.

[15] Sartorius N (2018). Depression and diabetes. Dialogues Clin Neurosci.

[16] Yang L, Jiang Y, Xu S, Bao L, Parker D, Xu X, Li J (2018). Evaluation of frailty status among older people living in urban communities by Edmonton frail scale in Wuhu, China: a cross-sectional study. Contemp Nurse.

[17] Carneiro JA, Lima CA, Costa FM, Caldeira AP (2019). Health care are associated with worsening of frailty in community older adults. Rev Saude Publica.

[18] Li CL, Stanaway FF, Lin JD, Chang HY (2018). Frailty and health care use among community-dwelling older adults with diabetes: a population-based study. Clin Interv Aging.

[19] García-Esquinas E, Graciani A, Guallar-Castillón P, López-García E, Rodríguez-Mañas L, Rodríguez-Artalejo F (2015). Diabetes and risk of frailty and its potential mechanisms: a prospective cohort study of older adults. J Am Med Dir Assoc.

